# Measurements of Serum Pituitary-Gonadal Hormones and Investigation of Sexual and Reproductive Functions in Kidney Transplant Recipients

**DOI:** 10.4061/2010/612126

**Published:** 2010-07-27

**Authors:** Guang-chun Wang, Jun-hua Zheng, Long-gen Xu, Zhi-lian Min, You-hua Zhu, Jun Qi, Qiang-lin Duan

**Affiliations:** ^1^Department of Urology, Shanghai Tenth People's Hospital, Tongji University, Shanghai 200072, China; ^2^Kidney Transplantation Center, 117th Hospital of PLA, Hangzhou 310013, China; ^3^Department of Urology, Changzheng Hospital, Second Military Medical University, Shanghai 200003, China; ^4^Department of Urology, Xinhua Hospital, Shanghai Jiao Tong University, Shanghai 200433, China

## Abstract

*Objective*. To investigate changes in serum pituitary-gonadal hormones and restoration of sexual and reproductive functions after successful kidney transplantation. *Patients and Methods*. Serum pituitary-gonadal hormones before and after kidney transplantation were measured in 78 patients with end-stage renal disease (ESRD) and in 30 healthy adults. Pre- and postoperative semen specimens of 46 male recipients and 15 male controls were collected and compared. Additional 100 married kidney transplant recipients without children were followed up for 3 years to observe their sexual function and fertility. *Results*. Serum PRL, LH, and T or E_2_ levels gradually restored to the normal ranges in all kidney transplant recipients, and sperm density, motility, viability, and morphology significantly improved in the male recipients 4 months after successful kidney transplantation (*P* < .05). Thirty-three male recipients (55.93%) reobtained normal erectile function, and 49 kidney transplant recipients (61.25%) had children within the 3-year follow-up period. *Conclusion*. Successful kidney transplantation could effectively improve pituitary-gonadal hormone disturbance and sexual and reproductive dysfunctions of ESRD patients.

## 1. Introduction

End-stage renal disease (ESRD) is usually associated with pituitary-gonadal hormone disturbance, and most ESRD patients are often perplexed by moderate or severe sexual dysfunction [1]. Although hemodialysis or peritoneal dialysis could prolong the survival time of ESRD patients significantly, it seems difficult to recover the impaired pituitary-gonadal axis function. Whether or not could sexual function be restored by successful kidney transplantation? Akbari et al. [2] found that serum testosterone (T) level remained low and luteinizing hormone (LH) level began increasing in most male recipients during hemodialysis, and that both T and LH levels restored to the normal range after successful kidney transplantation, implying that hemodialysis and kidney transplantation might have different effects in improving pituitary-gonadal axis dysfunction in ESRD patients. 

Sexual dysfunction and infertility are not uncommon in ESRD patients, and the occurrence of sexual dysfunction is about 50% to 70% in both male and female patients during dialysis [3]. According to the statistics, 30–40% male kidney transplant recipients are younger than 50 years, and sexual dysfunction and infertility are their major concerns before transplantation. However, there are few studies concerning serum pituitary-gonadal hormones and sexual dysfunction and infertility during dialysis and after kidney transplantation in Asian populations. The present paper investigated pre- and post-operative serum pituitary-gonadal hormones and sexual function and fertility in a Chinese population to see whether or not successful kidney transplantation could alleviate hormone disorders and improve sexual and reproductive functions.

## 2. Patients and Methods

### 2.1. Case Selection

Included in this study were 78 married patients with ESRD (46 males and 32 females) who ranged in age from 25 to 45 years with a mean of 33 years. The course of disease ranged from 2 to 6 years (mean 3.6 years), and the preoperative course of dialysis ranged from 3 to 34 months (mean 7.5 months). ABO and Rh blood types of the recipients and the donors were comparable, and the proportion of dead cells in lymphocytotoxicity test was <10%. Immunosuppressive therapy (Cyclosporine A (CsA) + Azathioprine (AZA) + Prednisone) was initiated from postoperative day 1. Additional 30 healthy adults (15 males and 15 females) with normal erectile function or menstrual cycles were selected from the family members (SCr <2.0 mg/dl) of the 78 patients as controls, who ranged in age from 26 to 45 years (mean 33 years). There was no significant difference in age, habits, and living environments between the study and control groups. 

Additional 100 married kidney transplant recipients (68 males and 32 females) without children who ranged from 26 to 45 years (mean 34 years) were followed up for 3 years. Exclusion criteria were (1) recipients who were unmarried, widowed, or divorced, (2) recipients who suffered from diabetes, hypertension, or other diseases that might significantly impair sexual or reproductive functions, and (3) recipients who had children before kidney transplantation. These recipients received the same immunosuppressive therapy.

All selected recipients in this study were from Shanghai Tenth People's Hospital, 117th Hospital of PLA and Changzheng Hospital. no organs were used from prisoners and no subjects were prisoners in this study. Written informed consents were obtained from all subjects involved. This study was approved by the local ethics committee and conducted in accordance with the Declaration of Helsinki (1964).

### 2.2. Methods

Serum pituitary-gonadal hormones were measured as follows: Venous blood samples of the 78 ESRD patients were collected during dialysis and at 1-2 and 3-4 months postoperatively, respectively. Blood samples of the 32 female recipients were collected 4-5 days after the menstrual period. Venous blood samples of the 30 healthy adults served as controls. Serum prolactin (PRL), follicle stimulating hormone (FSH), LH, and T or E_2_ were measured immediately by electrochemiluminescence immunoassay (ElecsysSystem, Roche Diagnositics GmbH, Mannheim, Germany) according to the manufacturer's instructions. All blood samples were measured in duplicate. Semen specimens of the 46 male ESRD patients were collected before kidney transplantation and 4 months after transplantation when their renal function was nearly restored to the normal range, and semen specimens of the 15 male healthy adults were used as the control. All semen specimens were analyzed and compared between the two groups. 

The investigation items of the additional 100 married kidney transplant recipients during the 3-year follow-up period include recording and analyzing the day of first nocturnal erection and sexual activity after transplantation, sexual desire, degree of erection, satisfaction with sexual activities, frequency of sexual activities, and international index of erectile function-5 (IIEF-5) score for the 68 male patients and the day of first menstruation and sexual activity after transplantation, satisfaction with sexual activities, frequency of sexual activities, pregnancy, and reproductive function for the 32 female patients.

### 2.3. Statistical Analyses

Statistical analyses were performed with SPSS version 13.0 statistical software. Pre- and post-operative serum pituitary-gonadal hormone levels, the volume of semen, and sperm viability, density, and morphology were compared between the study and control groups, using *t* test for independent samples. *P* < .05 was considered statistically significant.

## 3. Results

### 3.1. Pre- and Postoperative Serum Pituitary-Gonadal Hormone Levels

Preoperative serum PRL and LH levels of the 46 male ESRD patients were significantly higher, and serum T level was significantly lower than that of the 15 male controls (both *P* < .01). Serum PRL, LH, and T levels of the 46 male ESRD patients gradually restored to the normal range after transplantation, and 3-4 months after transplantation no significant difference in serum PRL, LH and T was observed between the two groups (Table 1).

Preoperative serum PRL, LH, and FSH levels of the 32 female ESRD patients were significantly higher, and serum E_2_ level was significantly lower than that of the 15 female controls (both *P* < .01). Serum PRL, LH, FSH, and E_2_ levels of the 32 female patients gradually restored to the normal range after transplantation, and 3-4 months after transplantation no significant difference in serum PRL, LH, FSH, and E_2_ levels was observed between the two groups (Table 2). 

### 3.2. Comparison of Pre- and Postoperative Semen Analyses

Normal semen quality is defined as sperm density >30 × 10^6^/ml, sperms that move forward >50%, and sperms in normal morphology >14%. Of the 46 male ESRD patients, 12 patients (26.09%) had severe oligospermia (sperm density was <5 × 10^6^/ml), 29 patients (63.04%) had mild or moderate oligospermia (sperm density was 5–30 × 10^6^/ml), and only 5 patients (10.87%) had normal sperm density before transplantation, and the proportion of sperms in normal morphology was only 2%–12%. Four months after transplantation, sperm density, motility, and viability as well as the proportion of sperms in normal morphology were significantly higher than those before transplantation (*P* < .05) (Table 3).

### 3.3. Sexual Function

Of the 100 married kidney transplant recipients without children, 59 male and 21 female recipients were followed up for more than 3 years, and the remaining 20 recipients lost followup by the end of this study.

#### 3.3.1. Basic Information of the 80 Recipients

**(1) Occupation**

Researchers and civil servants (*n* = 21, 26.25%), workers (*n* = 35, 43.75%), farmers (*n* = 22, 27.5%), and freelancers (*n* = 2, 2.5%).

**(2) Education**

University or above (*n* = 22, 27.5%), senior middle school (*n* = 31, 38.75%), junior middle school (*n* = 21, 26.25%), and primary school (*n* = 6, 7.5%).

**(3) Marital Age**

1–18 years (mean 7.65 years).

**(4) Fertility**

49 (61.25%) had children, and the remaining 31 (38.75%) had no children.

#### 3.3.2. Sexual Function

(1) The day of first postoperative nocturnal erection in 59 male recipients was averagely 45 days (2–180 days) after transplantation, and the first postoperative sexual activity occurred averagely 252 days (80–560 days) after transplantation. Pre- and post-operative IIEF-5 scores of the 59 male recipients are listed in Table 4. Four months after transplantation, 33 (55.93%) of the 59 male recipients who suffered from different degrees of erectile dysfunction before transplantation reobtained normal erectile function after transplantation and were satisfied with their sexual activities. The mean frequency of postoperative sexual activities was 4 times (1–10 times) monthly.

(2) The first postoperative menstruation of the 21 female recipients occurred averagely 38 days (5–90 days) after transplantation, and the first postoperative sexual activity occurred averagely 324 days (110–690 days) after transplantation. The mean frequency of postoperative sexual activities was 1.6 times (1–4 times) monthly.

#### 3.3.3. Fertility

Of the 80 recipients who were followed up for 3 years, 49 (61.25%) had children and the remaining 31 (38.75%) had no children. Of the 21 female recipients who were followed up for 3 years, 8 patients (38.10%) had children, 4 patients (19.05%) experienced spontaneous abortion during the first trimenon of pregnancy, 5 patients (23.81%) terminated their pregnancy because of edema-hypertension-proteinuria syndrome or gradual worsening of their renal function, and the remaining 4 patients (19.05%) had never conceived.

#### 3.3.4. Sexual Psychology

All recipients felt anxious about or even afraid of sexual activities after transplantation, and therefore the frequency of sexual activities was very low or even naught in the first year after transplantation. Of the 59 male recipients, 46 patients (77.97%) felt anxious about sexual activities, 28 patients (47.46%) were afraid of sexual activities, and 19 patients (32.20%) deemed that sexual activities were harmful to the kidney grafts, even though most of them tried their first postoperative sexual intercourse within the first year after operation. Of the 21 female recipients, 17 patients (80.95%) deemed that sexual activities were harmful to the grafts. This figure was significantly higher than that in the male recipients (*P* < .05). Our 3-year follow-up showed that 72 recipients (90.00%) believed that sexual activities were unsafe within one year after kidney transplantation.

### 3.4. Attitudes towards Their Recipient Spouses

Of the 80 recipients who were followed up for 3 years, 47 spouses (58.75%) were willing to take care of the recipients lifetime, 25 spouses (31.25%) were willing for a take care of the recipients for the time being, and the remaining 8 spouses (10.00%) were unwilling to take care of the recipients at all. Fifty-seven spouses (71.25%) believed that the recipients could be cured. Twenty-five patients (31.25%) had marital relationships as that before kidney transplantation; the marital relationship became worse in 43 patients (53.75%) after operation; 12 patients (15.00%) got divorced. Twenty-six recipient spouses (32.50%) had extramarital affairs, 23 spouses (28.75%) could tolerate no sexual activities with the recipients, and 7 spouses (8.75%) felt disgusted about sexual activities with the recipients and eventually left them.

## 4. Discussion

Serum T, FSH, LH, and PRL levels were abnormal in ESRD patients even during hemodialysis or peritoneal dialysis [3–5]. Previous studies [6] showed that serum T level remained low and LH level was elevated in most ESRD patients during hemodialysis, but these hormone profiles could be restored after successful kidney transplantation, indicating that dialysis and transplantation play different roles in restoring the pituitary-gonadal axis dysfunction in ESRD patients. Pietrzak et al. [7] reported that 68.1% of their female kidney transplant recipients had regular menstrual cycles and ovulatory cycles similar to healthy women. 

The pre- and post-operative hormone changes of the 78 ESRD patients in our study indicated that the pituitary-gonadal function was significantly improved after kidney transplantation. Serum T level in the male recipients and E_2_ level in the female recipients restored to the normal range 3-4 months after kidney transplantation. However, some immunosuppressive regimens such as sirolimus seemed to impair the improvement of gonadal function in kidney transplant recipients [8]. Fritsche et al. [9] conducted a case-control study to compare serum T, FSH, LH, and PRL levels in matched kidney transplant recipients treated with or without sirolimus and found that serum T level was lower FSH and LH were higher in sirolimus-treated recipients than those in nonsirolimus-treated controls, but there was no significant difference in serum PRL level between the two groups. Another study [10] reported that immunosuppressive drugs such as TOR-I (target of rapamycin inhibitors) might decrease serum T level and increase serum LH and FSH levels. In addition, TOR-I might disrupt spermatogenesis. Gonadal function could be improved by successful kidney transplantation, but on the other hand, it could also be impaired by immunosuppressive drugs. It remains controversial whether kidney transplantation could improve gonadal, sexual, and reproductive functions, especially in the long run. 

Sexual disorders are common in ESRD patients, and about 50% of them had erectile dysfunction during dialysis [11, 12], which may be caused by multiple factors including lower serum T level, autonomic neuropathy, vascular diseases, medications, deterioration of underlying diseases, and psychological stress. It has been recognized that low serum T level may be correlated with poor sexual desire and erectile dysfunction. The results of our study showed that serum T level, sexual activities, and IIEF-5 score improved markedly after transplantation, which was consistent with the finding of Lessan-Pezeshki and Ghazizadeh [13] and Shamsa et al. [14]. 

There is no recognized appropriate time for recipients to have the first sexual activity after kidney transplantation, although it mainly depends on the normal function of the renal grafts and the overall situation of the recipients. It was found in our study that the pituitary-gonadal function nearly restored to the normal range in most recipients four months after operation, suggesting that the first postoperative sexual activity could be attempted from four months postoperatively. However, most recipients in our series did not dare to have the first postoperative sexual activity until six months later; the frequency of sexual activity in the female recipients was less than that in the male recipients; the first day of postoperative sexual activity in the female recipients was also two months later than that in the male recipients, probably due to the misunderstanding that sexual activity was harmful to the grafts, especially in female recipients. It is therefore important to provide kidney recipients with timely psychological consultation, support, and treatment.

Male ESRD patients are usually associated with spermatogenesis dysfunction, eventually leading to infertility. Semen analysis showed that the volume of semen, sperm count and motility were decreased in these patients. Histological examination of the testes further confirmed that spermatogenesis was inactive in such patients [15]. We found that sperm density, motility, and viability as well as the proportion of sperms in normal morphology were significantly higher 4 months after transplantation than those before operation. However, kidney transplantation was unable to recover spermatogenesis in patients who suffered from uremia before or during adolescence, probably because this period is crucial for spermatogenesis [16].

With the development of kidney transplantation technique and the application of new immunosuppressants, pregnancy after kidney transplantation is no longer impossible [17–19]. In this study, 49 recipients (61.25%) had children, 8 female patients (38.10%) had children, and 9 female patients (42.86%) experienced spontaneous abortion. Timely medical consultation and support should be provided for female recipients who are prepared for pregnancy. As pregnancy would overload the function of the kidney grafts, special attention should be paid to prevent abortion, premature delivery, edema-hypertension-proteinuria syndrome, infection, and graft function and fetal abnormality during pregnancy. Based on previous reports [18, 19], the recommended inclusion criteria of pregnancy in kidney transplant female recipients are as follows: <30 years, SCr < 176.8 *μ*mol/L, no hyperlipemia, no hypertension or well-controlled hypertension, no proteinuria, good general conditions, no dilated renal calices as detected by B-ultrasound, and good tolerance with immunosuppressive therapy. 

In summary, with the development of social civilization, a new biopsychosocial medical model has been gradually recognized. Our study showed that serum pituitary-gonadal hormone levels were gradually restored to the normal range, and sexual and reproductive functions were also significantly improved after kidney transplantation, indicating that successful kidney transplantation could effectively improve pituitary-gonadal hormone disturbance and sexual and reproductive dysfunction in ESRD patients. Nevertheless, some recipients had a misunderstanding or psychologic barriers about postoperative sexual activity and fertility, and some other recipients were unable to get appropriate health care and psychological support from their spouses. It is therefore important and necessary to provide kidney graft recipients with timely medical education and psychological support after successful kidney transplantation.

## Figures and Tables

**Table 1:** Serum pituitary-gonadal hormone levels before and after transplantation in the 46 male ESRD patients and 15 male controls.

Item	*n*	PRL (ug/L)	LH (IU/L)	FSH (IU/L)	T (nmol/L)
Control	15	8.42 ± 0.94	14.00 ± 4.01	17.00 ± 3.12	23.50 ± 4.61
Preop.	46	36.80 ± 7.30*	30.68 ± 35.58*	18.30 ± 8.01	15.58 ± 3.84*
1-2 months postop.	46	15.80 ± 2.08	20.01 ± 12.68	17.42 ± 7.24	18.77 ± 4.18
3-4 months postop.	46	9.41 ± 1.12^†^	13.84 ± 6.72^†^	16.84 ± 6.72	22.94 ± 4.86^†^

*P < .01 versus control group; ^†^ *P* < .01 versus preoperation.

**Table 2:** Serum pituitary-gonadal hormone levels before and after transplantation in the 32 female ESRD patients and 15 female controls.

Item	*n*	PRL (ug/L)	LH (IU/L)	FSH (IU/L)	E_2_ (pmol/L)
Control	15	15.10 ± 3.02	12.01 ± 3.84	8.42 ± 3.80	131.5 ± 22.30
Preop.	32	24.60 ± 3.80*	37.23 ± 4.56*	16.92 ± 11.30*	76.0 ± 6.30*
1-2 months postop.	32	15.68 ± 2.04	21.61 ± 9.04	12.45 ± 6.30	242.35 ± 4.08
3-4 months postop.	32	16.30 ± 1.42^†^	13.04 ± 2.62^†^	10.10 ± 7.60^†^	124.50 ± 27.23^†^

*P < .01 versus control group; ^†^ *P* < .01 versus preoperation.

**Table 3:** Comparison of pre- and post-operative semen analyses between the study and control groups.

Item	*n*	Volume (ml)	Motility (%)	Survival rate (%)	Density (10^6^/ml)	Normal morphology (%)
control	15	3.3 ± 0.4	64.2 ± 3.4	80.5 ± 2.4	72.8 ± 11.2	15%–27%
preoperative	46	1.7 ± 0.5^†^	13.6 ± 3.0^†^	24.5 ± 3.6^†^	20.6 ± 6.3^†^	2%–12%^†^
4 months postop.	46	2.9 ± 0.6*	60.9 ± 4.7*	82.3 ± 4.7*	67.2 ± 13.5*	12%–25%*

*P < .05 versus preoperative group; ^†^ *P* > .05 versus control group.

**Table 4:** IIEF-5 score before and after kidney transplantation.

Item	*n*	IIEF-5 score				
		22–25	17–21	12–16	8–11	1–7
Preoperative	59	5	20	15	10	9
2 months postop.		16*	18	11	8	6
4 months postop.		38^†^	8^†^	6^†^	4^†^	3^†^

*P < .05, preoperation versus 2 months after operation; ^†^ *P* < .05, preoperation versus 4 months after operation.

## References

[1] Anantharaman P, Schmidt RJ (2007). Sexual function in chronic kidney disease. *Advances in Chronic Kidney Disease*.

[2] Akbari F, Alavi M, Esteghamati A (2003). Effect of renal transplantation on sperm quality and sex hormone levels. *British Journal of Urology International*.

[3] Bailie GR, Elder SJ, Mason NA (2007). Sexual dysfunction in dialysis patients treated with antihypertensive or antidepressive medications: results from the DOPPS. *Nephrology Dialysis Transplantation*.

[4] Framarino Dei Malatesta M, Rossi M, Rocca B (2007). Fertility following solid organ transplantation. *Transplantation Proceedings*.

[5] Doumouchtsis KK, Kostakis AI, Doumouchtsis SK (2008). The effect of sexual hormone abnormalities on proximal femur bone mineral density in hemodialysis patients and the possible role of RANKL. *Hemodialysis International*.

[6] Matuszkiewicz-Rowińska J, Skórzewska K, Radowicki S (2004). Endometrial morphology and pituitary-gonadal axis dysfunction in women of reproductive age undergoing chronic haemodialysis—a multicentre study. *Nephrology Dialysis Transplantation*.

[7] Pietrzak B, Cyganek A, Jabiry-Zieniewicz Z (2006). Function of the ovaries in female kidney transplant recipients. *Transplantation Proceedings*.

[8] Tondolo V, Citterio F, Panocchia N (2005). Gonadal function and immunosuppressive therapy after renal transplantation. *Transplantation Proceedings*.

[9] Fritsche L, Budde K, Dragun D, Einecke G, Diekmann F, Neumayer H-H (2004). Testosterone concentrations and sirolimus in male renal transplant patients. *American Journal of Transplantation*.

[10] Huyghe E, Zairi A, Nohra J, Kamar N, Plante P, Rostaing L (2007). Gonadal impact of target of rapamycin inhibitors (sirolimus and everolimus) in male patients: an overview. *Transplant International*.

[11] Özdemir C, Eryilmaz M, Yurtman F, Karaman T (2007). Sexual functioning after renal transplantation. *Transplantation Proceedings*.

[12] Grossman EB, Swan SK, Muirhead GJ (2004). The pharmacokinetics and hemodynamics of sildenafil citrate in male hemodialysis patients. *Kidney International*.

[13] Lessan-Pezeshki M, Ghazizadeh S (2008). Sexual and reproductive function in end-stage renal disease and effect of kidney transplantation. *Asian Journal of Andrology*.

[14] Shamsa A, Motavalli SM, Aghdam B (2005). Erectile function in end-stage renal disease before and after renal transplantation. *Transplantation Proceedings*.

[15] Yadav R, Mehta SN, Kumar A, Guleria S, Seenu V, Tiwari SC (2008). A prospective analysis of testicular androgenic function in recipients of a renal allograft. *International Urology and Nephrology*.

[16] Inci K, Duzova A, Aki FT (2006). Semen variables and hormone profiles after kidney transplantation during adolescence. *Transplantation Proceedings*.

[17] Mastrobattista JM, Gomez-Lobo, Society for Maternal-Fetal Medicine V (2008). Pregnancy after solid organ transplantation. *Obstetrics and Gynecology*.

[18] Midtvedt K, Bjorang O, Letting A-S (2007). Successful pregnancy in renal transplant recipient with previous known polyomavirus nephropathy. *Clinical Transplantation*.

[19] Kashanizadeh N, Nemati E, Sharifi-Bonab M (2007). Impact of pregnancy on the outcome of kidney transplantation. *Transplantation Proceedings*.

